# Assessment of performance for a key indicator of One Health: evidence based on One Health index for zoonoses in Sub-Saharan Africa

**DOI:** 10.1186/s40249-022-01020-9

**Published:** 2022-10-22

**Authors:** Han-Qing Zhao, Si-Wei Fei, Jing-Xian Yin, Qin Li, Tian-Ge Jiang, Zhao-Yu Guo, Jing-Bo Xue, Le-Fei Han, Xiao-Xi Zhang, Shang Xia, Yi Zhang, Xiao-Kui Guo, Kokouvi Kassegne

**Affiliations:** 1 Department of Infectious and Tropical Diseases, School of Global Health, Chinese Center for Tropical Diseases Research, Shanghai Jiao Tong University School of Medicine, Shanghai, 200025 People’s Republic of China; 2 One Health Center, Shanghai Jiao Tong University-The University of Edinburgh, Shanghai, 200025 People’s Republic of China; 3 National Institute of Parasitic Diseases at Chinese Center for Disease Control and Prevention (Chinese Center for Tropical Diseases Research), National Health Commission of the People’s Republic of China (NHC) Key Laboratory of Parasite and Vector Biology, WHO Collaborating Center for Tropical Diseases, Shanghai, 200025 People’s Republic of China

**Keywords:** One Health index, One Health performance, Zoonoses, Sub-Saharan Africa

## Abstract

**Background:**

Zoonoses are public health threats that cause severe damage worldwide. Zoonoses constitute a key indicator of One Health (OH) and the OH approach is being applied for zoonosis control programmes of zoonotic diseases. In a very recent study, we developed an evaluation system for OH performance through the global OH index (GOHI). This study applied the GOHI to evaluate OH performance for zoonoses in sub-Saharan Africa.

**Methods:**

The framework for the OH index on zoonoses (OHIZ) was constructed including five indicators, 15 subindicators and 28 datasets. Publicly available data were referenced to generate the OHIZ database which included both qualitative and quantitative indicators for all sub-Sahara African countries (n = 48). The GOHI algorithm was used to estimate scores for OHIZ. Indicator weights were calculated by adopting the fuzzy analytical hierarchy process.

**Results:**

Overall, five indicators associated with weights were generated as follows: source of infection (23.70%), route of transmission (25.31%), targeted population (19.09%), capacity building (16.77%), and outcomes/case studies (15.13%). Following the indicators, a total of 37 sub-Sahara African countries aligned with OHIZ validation, while 11 territories were excluded for unfit or missing data. The OHIZ average score of sub-Saharan Africa was estimated at 53.67/100. The highest score was 71.99 from South Africa, while the lowest score was 40.51 from Benin. It is also worth mentioning that Sub-Sahara African countries had high performance in many subindicators associated with zoonoses, e.g., surveillance and response, vector and reservoir interventions, and natural protected areas, which suggests that this region had a certain capacity in control and prevention or responses to zoonotic events.

**Conclusions:**

This study reveals that it is possible to perform OH evaluation for zoonoses in sub-Saharan Africa by OHIZ. Findings from this study provide preliminary research information in advancing knowledge of the evidenced risks to strengthen strategies for effective control of zoonoses and to support the prevention of zoonotic events.

**Supplementary Information:**

The online version contains supplementary material available at 10.1186/s40249-022-01020-9.

## Background

One Health (OH) is an integrated and unifying approach that aims to sustainably balance and optimize the health of people, animals and ecosystems. Hence, OH performance index is referred to as the capacity to prevent or respond to humans, animals, and the environment health threats [1]. The OH approach implies multidisciplinary efforts with common goals to achieve better public health outcomes by helping with disease prediction, prevention, and preparedness at the interface between humans, animals, and their environments [2].

In 2018, three major international organizations, the World Health Organization (WHO), the United Nations’ Food and Agriculture Organization (FAO), and the World Organization for Animal Health (OIE), put the OH vision into practice by consolidating a formal partnership and strengthening their joint action to combat human-animal-environment health risks [3]. This culminated with the FAO-OIE-WHO (tripartite) zoonoses guide, titled “Taking A Multisectoral, One Health Approach: A Tripartite Guide to Addressing Zoonotic Diseases in Countries” (2018 TZG), which provides principles and best practices to assist countries in achieving sustainable and functional collaboration at the human-animal-environment interface [4]. In May 2021, the OH High-Level Expert Panel (OHHLEP) was launched to address the emergence and spread of zoonotic diseases [5]. The panel aims to advise four international organizations—the FAO, OIE, United Nations Environment Programme (UNEP), and the WHO—on the development of a long-term global plan of action to avert outbreaks of diseases. To that end, 26 international experts have been appointed to kickstart the OHHLEP, followed by a joint tripartite (FAO, OIE, WHO) and UNEP statement that advocates for mainstream OH so that they are better prepared to prevent, predict, detect, and respond to global health threats and promote sustainable development [6, 7].

Zoonoses are infections that are naturally transmitted between human beings and other vertebrates and can spread from food, water or the environment directly. Zoonoses alone represent 60% of world known infectious diseases, with a high proportion (70%) of pathogens coming from wildlife hosts [8]. With the acceleration of globalization, zoonotic emerging and re-emerging infectious diseases seriously harm human and health, husbandry development, and food security [9]. Throughout history, several epidemics and pandemics have been associated with zoonotic origins, with rapid spatial and temporal spread worldwide. These include, but are not limited to, the bubonic plague in the fourteenth century, the 1918 influenza pandemic, acquired immune deficiency syndrome (AIDS) since 1959, severe acute respiratory syndrome (SARS) in 2003, Middle East respiratory syndrome (MERS) in 2012, and the novel coronavirus disease (COVID-19) in 2019 [10]. According to pathogen types, zoonoses are classified as bacterial zoonoses, e.g., tuberculosis and brucellosis; viral zoonoses, e.g., AIDS and rabies; helminth zoonoses, e.g., schistosomiasis and echinococcosis; protozoan zoonoses, e.g., malaria and leishmaniasis; fungous zoonoses; rickettsia zoonoses; chlamydia zoonoses; mycoplasmosis; and exceptions, such as mad cow disease [11]. Zoonoses have different ways of transmission, including animals bite or scratch, by air, aerosol or dust particles, sexual contact or mother-to-child transmission, and other ways including oral transmission, animal or environmental indirect transmission [10]. Severe zoonoses are threatening to life security, public health and economic construction globally. For example, tuberculosis, leishmaniasis, and echinococcosis are major zoonotic diseases with high prevalence and disability-adjusted life years (DALYs), which were 1,829,729,478 and 47,030,118 for tuberculosis, 4,575,092 and 696,703 for leishmaniasis, and 900,005 and 122,457 for cystic echinococcosis, respectively, according to the global burden diseases (GBD) report in 2019.

The development degree of a country or area has great significance for its governance capacity of zoonoses. Developed countries have huge advantages in medical treatment, public health, economic construction, scientific research input and social welfare that most developing settings lack [9]. Sub-Saharan Africa has long been regarded as a low-economy region with low- and middle-income countries. This would have been reflected in weak response capacity/ability to zoonotic events [12, 13]. In addition, global climate change, deforestation, and poor animal husbandry methods accelerate risks for zoonotic diseases, especially in sub-Saharan African settings [14, 15]. According to the GBD report, in 2020, sub-Saharan Africa alone recorded point prevalence and DALYs of 257,082,412 and 17,547,387, respectively, for tuberculosis and were estimated at 168 633,396 and 43,197,058, respectively, for malaria.

One Health initiative on zoonoses, including governance capacity in surveillance and research activities, has been carried out in many countries/territories across the African continent [16]. In Kenya and Uganda, a global disease detection division [17] and a mutidisciplinary platform [18], respectively, have been established for zoonoses control and prevention under the OH approach. In the Horn of Africa (Ethiopia, Kenya, Uganda, Sudan, South Sudan, Somalia, Djibouti and Eritrea) and in Chad, Côte d'Ivoire, and Mali, international cooperation on the OH approach has been established for capacity building to support zoonoses control and prevention [19, 20]. However, such activities lack efficient interdepartmental collaboration mechanisms, or few outcomes are adequate to be implemented in local communities [18, 21].

In this study, we formulated indicators for zoonoses and applied OH principles [22, 23] to data retrieved from publicly available repositories to systematically analyze the OH index for zoonoses in sub-Saharan Africa. In addition, five major zoonotic diseases of public health importance worldwide such as tuberculosis, COVID-19, echinococcosis, leishmaniasis, and rabies [24], were selected as case studies for assessment. Findings from these aforementioned studies suggest imperative needs of the OH approach not only to consolidate existing achievements but also to implement integrative strategies in the control programmes of zoonotic events in sub-Saharan Africa.

## Methods

**Fig. 1:**
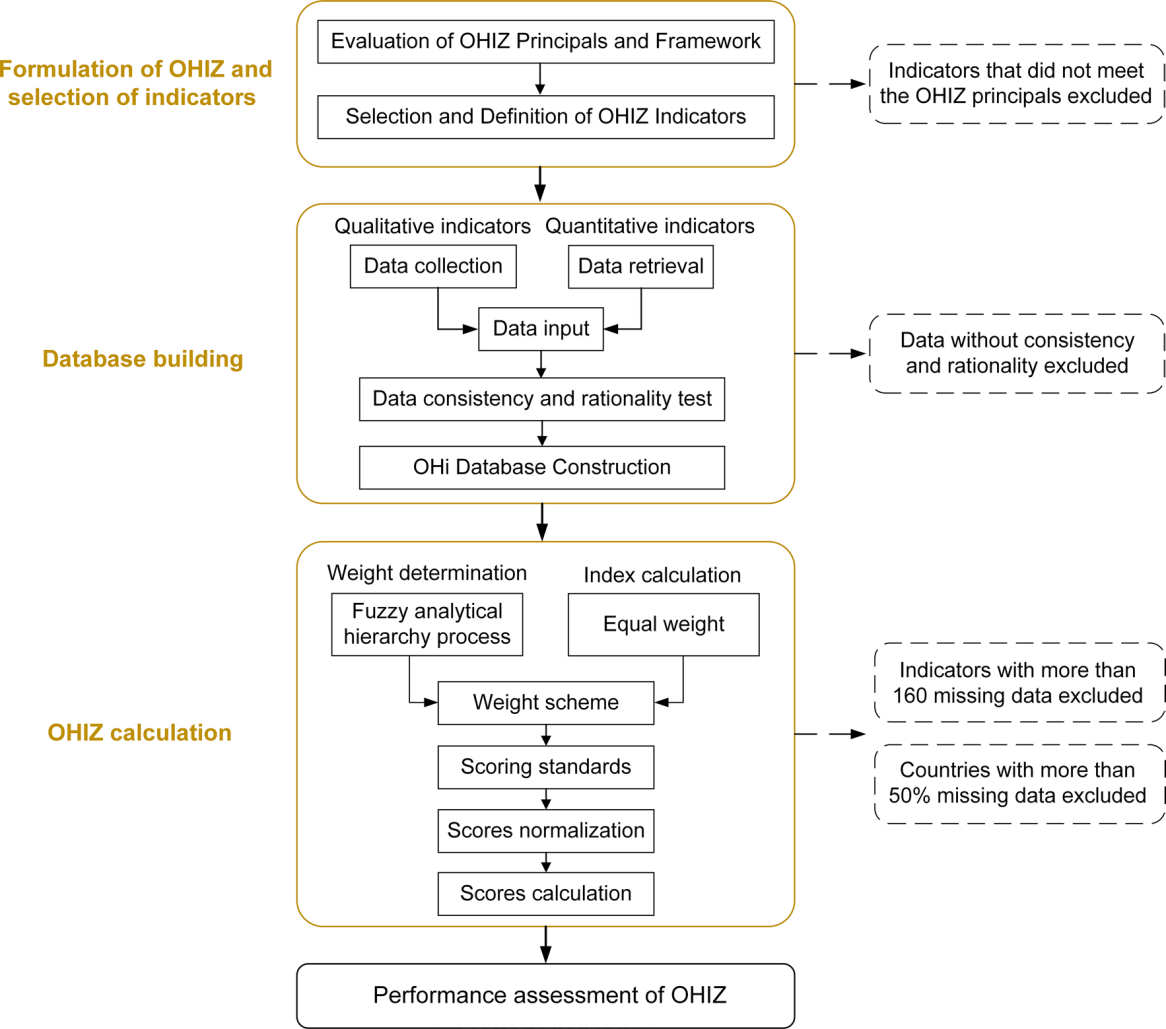
Flow chart for the processes involved in the assessment of the One Health performance for zoonoses.* OHIZ* One Health index on zoonoses,* OHi* One Health index

OH principles were applied to evaluate OH performance for zoonoses in sub-Saharan Africa. This study defines the OH index on zoonoses (OHIZ) as an indicator to assess the capacity of a country/territory to respond to or prevent zoonotic events associated with the holistic health of the human-animal-environmental interface. Figure 1 shows the schematic of the construction steps of OH performance assessment for zoonoses, which included formulation of OHIZ, selection of indicators, database building, and OHIZ calculation.

### Formulation of OHIZ and selection of indicators associated with zoonoses

Selection of the OHIZ database for zoonoses was based on seven principles as reported by Zhang and colleagues [23]. These data met the following selection criteria: fit to corresponding indicators of zoonoses; originated from authoritative sources with global or local zoonotic data; is available from public open sources with clear method of collecting; cover a sufficient number of countries/territories; cover recent temporal period and are updated annually; are measured with an established and unified method and peer-reviewed across countries/territories for single indicators; describe the status of zoonoses in the indicators at country-level.

Accordingly, three elements (indicators) of infectious diseases were included in the OHIZ framework and were referred to as the source of infection, route of transmission, and targeted population. Given that the OH approach covers areas of policy support, scientific research, and infrastructure construction, an indicator termed capacity building was also set. A further indicator referred to as outcomes (case studies) was added to form a five-indicator panel for OHIZ. Indicators that did not meet the OHIZ principles were excluded. Subindicators were conceived following the abovementioned indicators and information from previous studies [10, 11, 25, 26]. Zoonotic diseases of public health importance, e.g., tuberculosis, echinococcosis, leishmaniasis, and rabies were selected and were associated with the outcome indicator as cases studies. In addition, COVID-19, a newly emerging zoonotic disease of likely bat origin that has a huge impact on humanity and the environment potentially [9, 10], were also selected. The panel of indicators framework was thereafter developed into a set of 15 subindicators (Table 1).

**Table 1:** List of all-level OHIZ indicators and datasets alongside their weights

Indicator	Weight (%)	Subindicator	Weight (%)	Dataset	Weight (%)
Source of infection	23.70	Strategy and regulation	41.32	National guideline for surveillance/control	35.00
				National legislation on animal reservoirs	35.00
				Zoonoses capacity score	30.00
		Surveillance and response	33.36	General surveillance	33.33
				Vector control	33.33
				Wildlife reservoirs control	33.33
		Sanitation	25.32	Basic sanitation services	100
Route of transmission	25.31	Detection	45.15	Laboratory testing for zoonotic reservoirs (vectors and animals)	100
		Vector and reservoir interventions	54.85	Policy adoption of insecticide-treated mosquito nets	33.33
				Policy adoption of indoor residual spraying	33.33
				Prevention chemotherapy coverage of zoonoses	33.33
Targeted population	19.09	Vaccination regulation	28.98	Vaccination strategy and regulation vaccination	100
		Population coverage and cost of interventions	39.43	Proportion of population having basic drinking water and sanitation facilities Costs directed to chemotherapy/vaccination of humans	50.00
					50.00
		Inhabitants below 5 m above sea level	31.60	Number of inhabitants below 5 m above sea level	100
Capacity building	16.77	Health promotion for zoonoses	56.86	Legislation of zoonosis educational activities	16.66
				Prevention and control of zoonoses	16.66
				National plan for zoonoses vaccine	16.66
				Zoonotic events and human-animal interface	16.66
				Early warning for zoonoses	16.66
				Emergency/surveillance system	16.66
		Natural protected areas	43.14	Proportion of natural protected areas	100
Outcomes (case studies)	15.13	Cases of COVID-19	25.42	Infections number of COVID-19	50.00
				Vaccination coverage for COVID-19	50.00
		HD of echinococcosis	15.84	Echinococcosis DALYs	100
		HD of leishmaniasis	15.52	Leishmaniasis DALYs	100
		HD of rabies	20.33	Rabies DALYs	100
		HD of tuberculosis	22.88	Tuberculosis DALYs	100

*HD* Human DALYs; *DALYs* Disability-adjusted life years, *OHIZ* One Health index on zoonoses

### Building of the OHIZ database

The panel of indicators was classified into qualitative and quantitative indicators according to the database sets that were consulted. Data collection was applied to qualitative indicators, while data were retrieved for quantitative indicators [22, 23, 27]. During OHIZ data collection, qualitative data were labeled “0” for “data not found” and “1” for “data found”.

OHIZ database building referred to internationally published authoritative databases. A total of 28 comprehensive sets of OHIZ data were identified, including 13 datasets retrieved from the WHO database [28], three datasets from the OIE-WHAIS and the FAO-Emergency Prevention System for Animal Health (EMPRES) database [29], four datasets from the World Bank (WB) database [30], four from the global health security (GHS) index [31], and four from the GBD database of global health data exchange (GHDx) [32, 33]. Details on the data sources are listed in Additional file 1. After the OHIZ database was generated, all data were checked for consistency and rationality, and unfit data were excluded.

### Calculation and validation of OHIZ

The OHIZ algorithm from the robust global One Health index (GOHI) algorithm system that was reported recently [22, 23], was used to estimate OHIZ. Indicator weights were determined by adopting the fuzzy analytical hierarchy process (FAHP) [34], followed by fuzzy comparison matrix formation [22, 23] (Additional file 1). For indicators with values of “0” or “1”, appropriate measures were taken to correct bias from overpolarization.

**Fig. 2:**
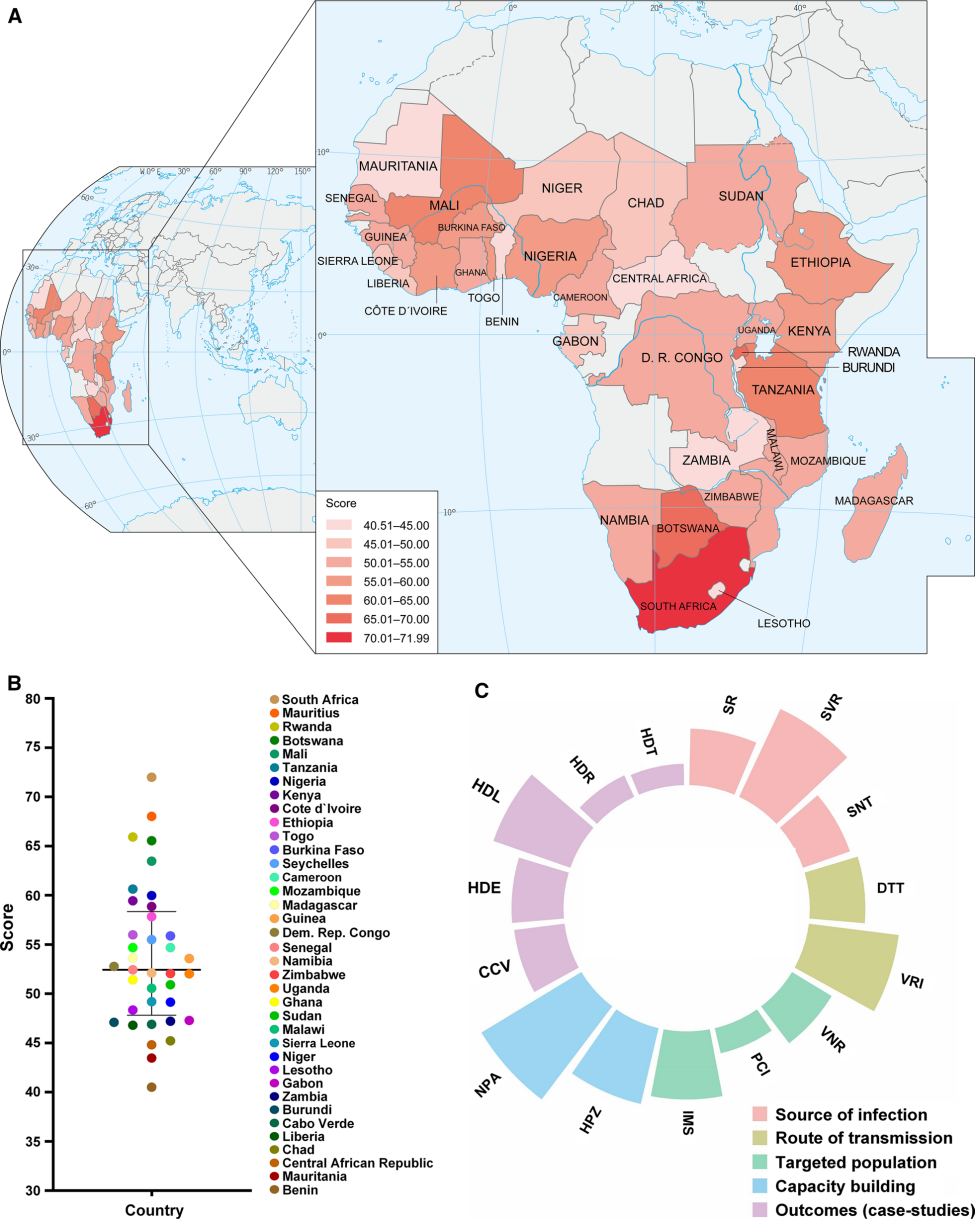
OHIZ overall scores of sub-Sahara African countries. **A** Sub-Sahara African countries OHIZ map. **B** OHIZ scores of sub-Sahara African countries. Data statistics included 37 sub-Sahara African countries. **C** Scores trend chart of sub-Sahara African countries for all-level indicators of OHIZ. Subindicators are denoted by standing initial as follows: *SR* strategy and regulation, *SVR* surveillance and response, *SNT* sanitation, *DTT* detection, *VRI* vector and reservoir interventions, *VNR* vaccination regulation, *PCI* population coverage and cost of interventions, *IMS* inhabitants below 5 m above sea level, *HPZ* health promotion for zoonoses, *NPA* natural protected areas, *CCV* cases of COVID-19, *HDE* human DALYs of echinococcosis; *HDL* human DALYs of leishmaniasis, *HDR* human DALYs of rabies, *HDT* human DALYs of tuberculosis. *OHIZ* One Health index on zoonoses

According to the WB classification criteria for countries and regions, there are 47 countries/territories in sub-Saharan Africa. OHIZ was analyzed for all sub-Sahara African countries (n = 47). Criteria of data of the same indicators from three similar countries were used to exclude biased data or countries with missing data. When there were more than 160 missing data points for an indicator, the indicator was excluded. When there were more than 50% missing data for a country, the country was excluded from the final list. A total of 37 sub-Sahara African countries were retained for the OHIZ (Fig. 2).

## Results

### OHIZ indicators and datasets

This study identified 28 datasets for zoonoses under 15 subindicators and five OHIZ indicators, which were all associated with weights (Table 1). Among the five indicators, route of transmission scored the highest (25.31%) weight, followed by source of infection (23.70%), targeted population (19.09%), and capacity building (16.77%). Outcomes (case studies) accounted only 15.13%. Weight for subindicators was also estimated according to the weight calculation. For example, in the source of infection, the strategy and regulation subindicator weighted 41.32%; in the route of transmission, the vector and reservoir interventions subindicator was estimated at 54.85%; in the targeted population, the population coverage and cost of interventions subindicator weighted 39.43%; in capacity building, the subindicator of health promotion for zoonoses was 56.86%; and in outcomes (case studies), the COVID-19 subindicator weighted 25.42%, out of three, two, three, two, and five subindicators under each of the indicators, respectively. Meanwhile, the weights for the datasets were attributed on average (Table 1).

### Sub-Saharan Africa scores for OHIZ

A total of 37 sub-Sahara African countries qualified for OHIZ score evaluation, after 10 countries that did not meet the inclusion criteria were excluded. Figure 2A shows the 37 countries/territories with different shades of red color, which reflected ranges of scores. The Country scores for subindicators are shown in Fig. 2B, C. The average score of sub-Sahara African countries was 53.67, with better scores in subindicators of surveillance and response, vector and reservoir interventions, natural protected areas, and leishmaniasis control. South Africa had the highest score (71.99), suggesting that the country has a strong capacity in responding to or preventing zoonotic events. South Africa, Mauritius, Rwanda, Botswana, Mali, Tanzania, Nigeria, Kenya, Cote d’Ivoire, and Ethiopia were the top 10 countries that had better OH performance for zoonoses, while Benin had the lowest score (40.51) (Table 2).

**Table 2:** Indicator scores of OH performance on zoonoses in sub-Saharan Africa. Ranks included 37 sub-Sahara African countries

Country	Zoonoses		Source of infection		Route of transmission		Targeted population		Capacity building		Outcomes (case studies)	
	Score	Rank	Score	Rank	Score	Rank	Score	Rank	Score	Rank	Score	Rank
South Africa	71.99	1	89.03	1	77.66	2	67.30	2	67.75	31	46.45	3
Mauritius	68.02	2	80.14	2	61.47	20	62.24	3	66.34	34	69.13	1
Rwanda	65.93	3	68.80	6	72.30	7	59.25	5	82.17	12	41.18	7
Botswana	65.55	4	77.03	3	64.66	18	60.35	4	76.39	21	43.62	5
Mali	63.46	5	69.07	5	60.31	21	70.39	1	86.78	5	25.40	31
Tanzania	60.64	6	61.00	11	68.12	13	50.74	10	79.95	15	38.64	12
Nigeria	59.97	7	62.72	9	65.86	17	53.84	9	76.46	20	35.26	20
Kenya	59.45	8	58.14	14	74.20	5	57.09	8	71.19	27	26.84	30
Cote d’Ivoire	58.86	9	65.15	7	71.89	8	46.52	11	68.12	30	32.55	23
Ethiopia	57.83	10	49.12	27	66.86	16	57.48	7	88.58	3	22.77	34
Togo	55.98	11	47.13	28	80.15	1	23.16	29	85.07	7	38.59	13
Burkina Faso	55.88	12	54.94	18	74.86	4	35.42	16	83.94	8	20.31	36
Seychelles	55.50	13	73.29	4	41.77	33	25.13	26	83.38	10	58.01	2
Cameroon	54.69	14	54.80	19	63.39	19	38.41	14	82.70	11	29.49	27
Mozambique	54.68	15	55.04	16	70.31	9	17.65	34	90.03	2	35.51	19
Madagascar	53.63	16	60.71	12	56.96	24	27.16	24	81.28	13	39.75	11
Guinea	53.58	17	55.41	15	67.46	14	21.94	30	86.79	4	30.65	25
Dem. Rep. Congo	52.78	18	61.70	10	41.41	34	57.50	6	76.88	19	25.18	32
Senegal	52.44	19	62.95	8	69.79	10	24.82	27	62.84	36	30.25	26
Namibia	52.12	20	53.41	21	52.73	27	34.36	17	80.42	14	40.14	9
Zimbabwe	52.06	21	51.17	24	74.09	6	15.29	36	74.89	23	37.68	15
Uganda	52.02	22	44.37	30	77.60	3	27.27	23	73.52	26	28.62	29
Ghana	51.42	23	55.01	17	55.59	26	33.93	18	77.86	18	31.57	24
Sudan	50.91	24	54.08	20	42.12	32	36.37	15	85.69	6	40.47	8
Malawi	50.56	25	51.59	23	47.95	31	42.43	13	74.73	24	36.80	16
Sierra Leone	49.19	26	42.09	35	56.60	25	21.10	31	90.23	1	37.85	14
Niger	49.15	27	49.81	26	69.45	11	27.58	21	67.23	32	21.32	35
Lesotho	48.35	28	44.61	29	52.52	28	30.72	20	79.64	17	34.81	22
Gabon	47.28	29	58.38	13	41.18	35	27.50	22	64.46	35	46.02	4
Zambia	47.21	30	50.63	25	57.16	22	23.68	28	75.49	22	23.52	33
Burundi	47.10	31	42.59	34	67.35	15	19.72	33	70.51	28	28.92	28
Cabo Verde	46.89	32	52.93	22	33.79	37	33.33	19	79.83	16	39.93	10
Liberia	46.80	33	33.45	36	38.82	36	45.48	12	83.63	9	41.90	6
Chad	45.22	34	43.89	33	68.96	12	25.29	25	57.75	37	18.86	37
Central African Rep	44.82	35	44.17	32	49.30	30	20.96	32	74.13	25	35.99	17
Mauritania	43.47	36	44.19	31	52.07	29	15.90	35	68.29	29	35.21	21
Benin	40.51	37	27.66	37	57.01	23	15.29	37	66.76	33	35.76	18

### Indicator scores of OHIZ in the sub-Sahara African countries

**Fig. 3:**
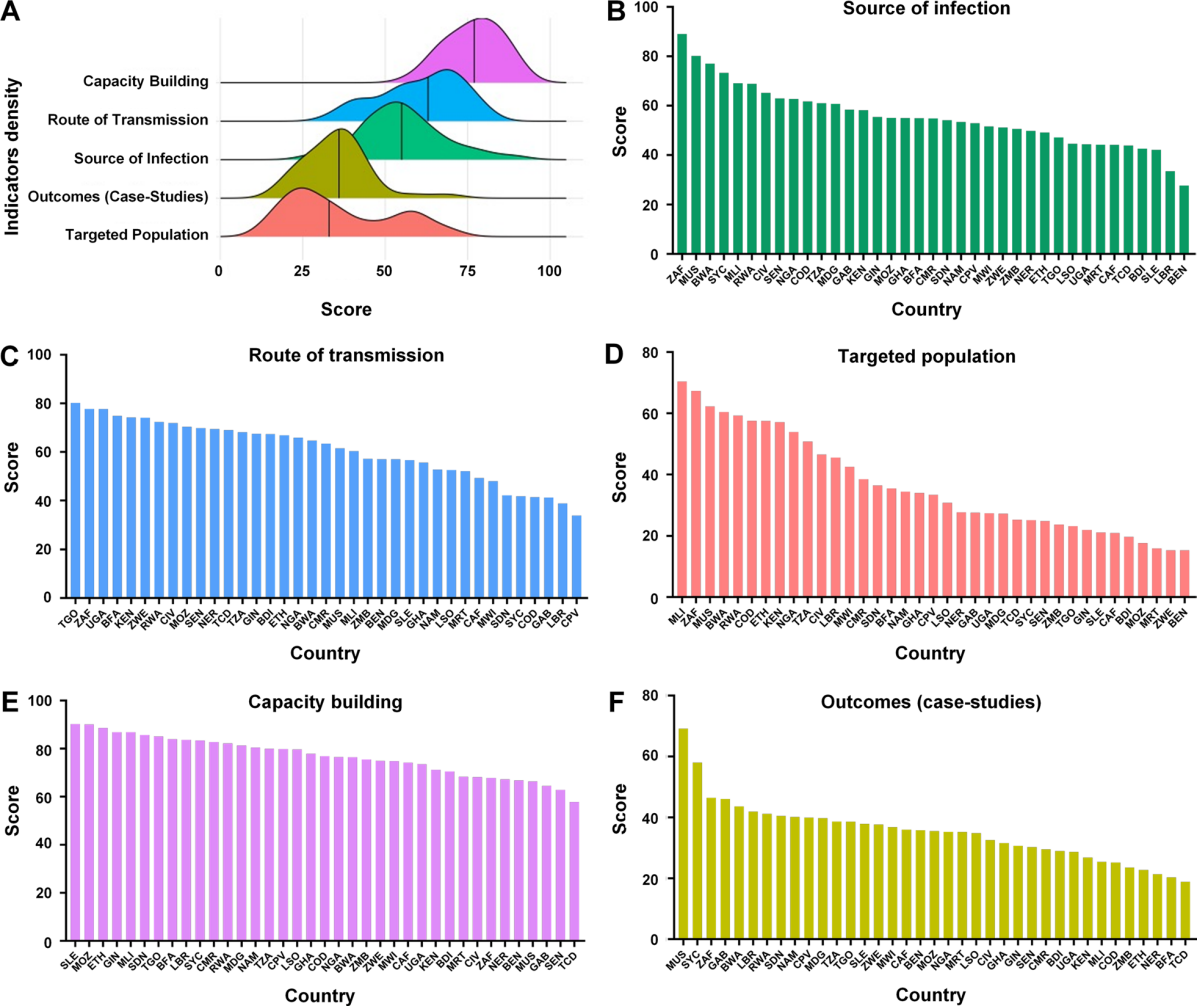
OHIZ scores density of sub-Sahara African countries. **A** Score density of OHIZ indicators in sub-Saharan Africa. **B**–**F** Scores of OHIZ indicators across sub-Saharan Africa. Sub-Sahara African countries were ranked from left to right according to indicator scores. *ZAF* South Africa, *MUS* Mauritius, *RWA* Rwanda, *BWA* Botswana, *MLI* Mali, *TZA* Tanzania, *NGA* Nigeria, *KEN* Kenya, *CIV* Cote d’Ivoire, *ETH* Ethiopia, *TGO* Togo, *BFA* Burkina Faso, *SYC* Seychelles, *CMR* Cameroon, *MOZ* Mozambique, *MDG* Madagascar, *GIN* Guinea, *COD*0 Democratic Republic of Congo, *SEN* Senegal, *NAM* Namibia, *ZWE* Zimbabwe, *UGA* Uganda, *GHA* Ghana, *SDN* Sudan, *MWI* Malawi, *SLE* Sierra Leone, *NER* Niger, *LSO* Lesotho, *GAB* Gabon, *ZAM* Zambia; *BDI* Burundi, *CPV* Cabo Verde, *LBR* Liberia, *TCD* Chad, *CAF* Central African Republic, *MRT* Mauritania, *BEN* Benin. *OHIZ* One Health index on zoonoses

Sub-Sahara African countries performed best in the indicator of capacity building for zoonoses (76.80 ± 8.25), overall. The indicator scores obtained for the route of transmission, source of infection, and outcomes (case studies) were 60.64 ± 12.43, 55.41 ± 12.52, and 35.27 ± 10.03, respectively (Fig. 3A). The OHIZ score for the indicator of the targeted population (33.33 ± 28.87) was the lowest, overall, while it was not normally distributed across the region. Following the indicator scores, South Africa had the highest score (89.03), while Benin received the lowest performance capacity (27.66) in responding to or preventing the source of infection (Fig. 3B). For performance capacity in responding to or preventing the route of transmission, the highest score was by Togo (81.15), and the lowest score was achieved by to Cabo Verde (33.79) (Fig. 3C). Mali scored highest (70.39) in capacity for the targeted population indicator, while Benin scored lowest (15.29) (Fig. 3D). For performance in capacity building for zoonoses, Sierra Leone ranked first (90.23), while the lowest score was by Chad (57.75) (Fig. 3E). In terms of outcomes (case studies) for zoonoses, Mauritius had the highest performance (69.13) in responding to or preventing tuberculosis, COVID-19, echinococcosis, leishmaniasis, and rabies collectively, whereas the Chad ranked lowest (18.86) (Fig. 3F). Details are provided in Table 2.

### Subindicator scores of the OHIZ in the sub-Sahara African countries

**Fig. 4:**
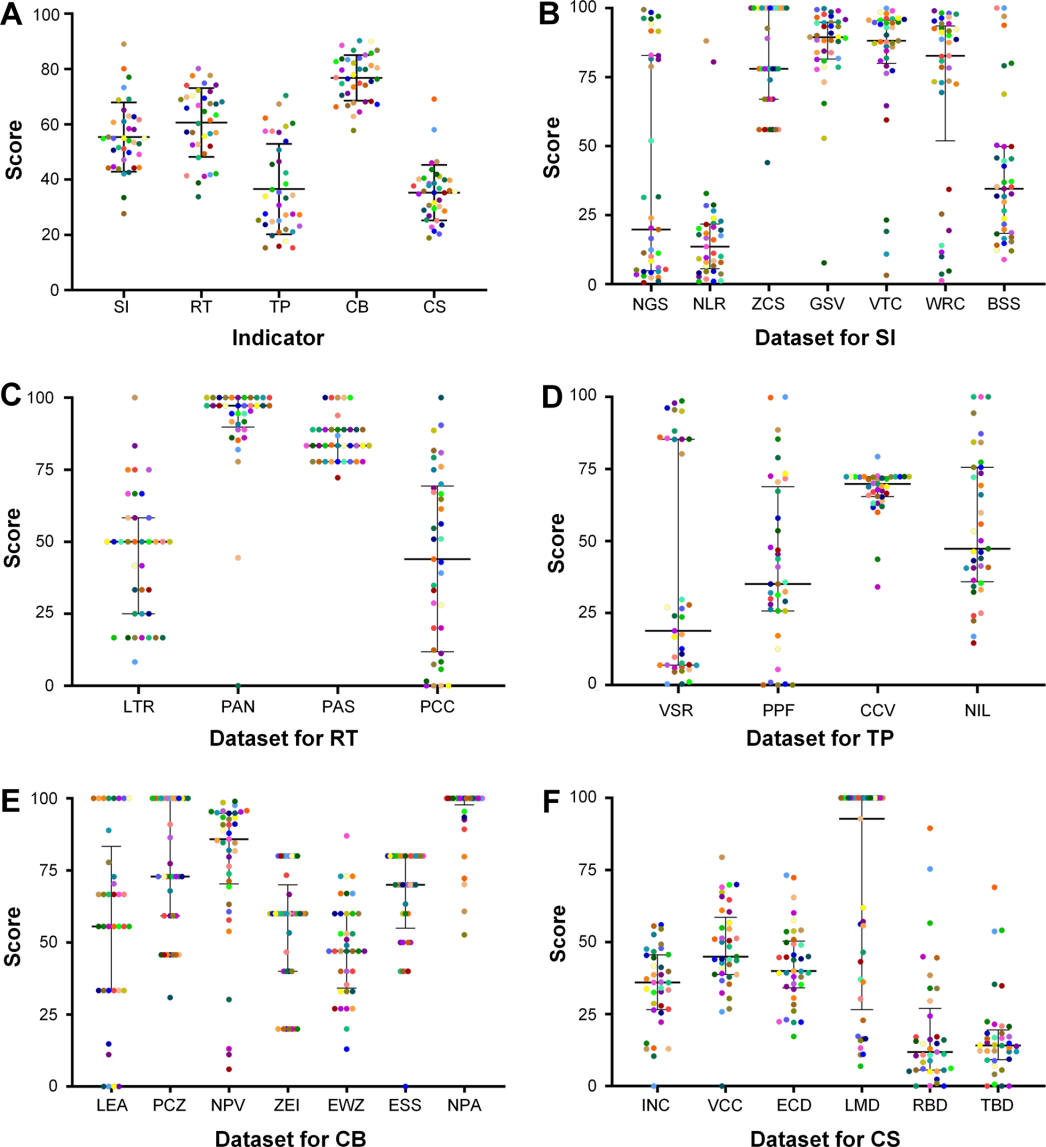
Dataset scores of OHIZ in sub-Sahara African countries. **A** Score scatter of OHIZ indicators. Data statistics included 37 sub-Sahara African countries. Indicators are denoted by standing initial as follows: SI, source of infection; RT, route of transmission; TP, target population; CB, capacity building; CS, outcomes (case studies). **B**–**F** Score scatter of OHIZ datasets. Data statistics included 37 sub-Sahara African countries. Datasets are denoted by standing initial as follows: *NGS* National guideline for surveillance/control, *NLR* National legislation on animal reservoirs, *ZCS* Zoonoses capacity score, *GSV* General surveillance; *VTC* Vector control, *WRC* Wildlife reservoirs control, *BSS* Basic sanitation services, *LTR* Laboratory testing for zoonotic reservoirs (vectors and animals), *PAN* Policy adoption of insecticide-treated mosquito nets; *PAS* Policy adoption of indoor residual spraying, *PCC* Prevention chemotherapy coverage of zoonoses, *VSR* Vaccination strategy and regulation vaccination, *PPF* Proportion of population having basic drinking water, *CCV* Costs directed to chemotherapy/vaccination of humans, *NIL* Number of inhabitants below 5 m above sea level, *LEA* Legislation of zoonosis educational activities, *PCZ* Prevention and control of zoonoses; *NPV* National plan for zoonoses vaccine, *ZEI* Zoonotic events and human-animal interface, *EWZ* Early warning for zoonoses; *ESS* Emergency/surveillance system, *NPA* Proportion of natural protected areas, *INC* Infections number of COVID-19, *VCC* Vaccination coverage for COVID-19, *ECD* Echinococcosis DALYs, *LMD* Leishmaniasis DALYs, *RBD* Rabies DALYs, *TBD* Tuberculosis DALYs. *OHIZ* One Health index on zoonoses

The average capacity in strategy and regulation to respond to and prevent sources of infection for sub-Sahara African countries was 39.55 for national guidelines for surveillance/control, while it was 17.24 and 80.64 for national legislation on animal reservoirs and zoonoses capacity scores, respectively. In addition, the average scores of sub-Sahara African countries in surveillance and response to source of infection were 85.83 for general surveillance, 80.84 for vector control, and 69.59 for wildlife reservoirs control, while in sanitation, the average capacity was estimated at 40.32 for basic sanitation services (Fig. 4B).

The average score for detection in responding to and preventing the route of transmission for laboratory testing for zoonotic reservoirs (vectors and animals) in sub-Sahara African countries was 45.05. The average score for policy adoption of insecticide-treated mosquito nets was 90.93, that for policy adoption of indoor residual spraying was 85.68 and that for prevention chemotherapy coverage of zoonoses was 43.81 in terms of capacity of vector and reservoir interventions (Fig. 4C).

Sub-Saharan Africa scored 67.46 for costs directed to chemotherapy/vaccination of humans and 41.79 for proportion of population having basic drinking water and sanitation facilities in terms of capacity performance for population coverage and cost of interventions, while the average score was 37.52 for vaccination strategy and regulation vaccination in capacity of vaccine for target population (Fig. 4D).

The average scores of sub-Sahara African countries in capacity building were 57.42 for legislation of zoonosis educational activities, 75.49 for zoonoses prevention and control, 77.35 for zoonoses vaccine national plan, 54.59 for zoonotic events and the human-animal interface, 48.39 for zoonotic early warning, and 65.77 for emergency/surveillance system in health promotion for zoonoses, while it was 94.77 for proportion of natural protected areas in terms of natural protected areas building (Fig. 4E).

The OH index for the five zoonotic diseases that were assessed in this study, revealed the highest score of human DALYs of leishmaniasis (66.15), followed by those of echinococcosis (42.20), rabies (19.19), and tuberculosis (17.31). However, the capacity of sub-Sahara African countries in responding to and preventing COVID-19 scored an average vaccination coverage estimated at 47.69 and that of infectious number at 34.56 (Fig. 4F).

## Discussion

This study used a newly established evaluation system, GOHI, to assess OH performance for zoonoses through scores of indicators, and provided essential guidance and references for zoonotic event prevention and control in sub-Saharan Africa.

The OHIZ datasets built in this study referred to relatively complete data for zoonoses, from international organizations and authoritative databases, such as WHO, OIE-WHAIS, FAO-EMPRES, WB, GHS, and GBD. Following the very recently developed assessment tool for OH performance [22, 23], we used indicators based on guidelines for OH and zoonoses, and generated OHIZ datasets that fit to research approaches for zoonoses from a global and holistic view.

The algorithm used for OHIZ in this study referred to the GOHI algorithm system [34], which provided scores for 37 sub-Sahara African countries out of the 48. A total of 11 countries/territories excluded from this study were of low quality or presented insufficient data for OHIZ calculation [23]. However, such an exclusion suggests that publicly available data that would reflect application of the OH approach for zoonoses control and prevention are needed.

Throughout the OHIZ scores identified by this study, only South Africa exceeded 70 in scores (71.99). In addition, five countries exceeded 60 in scores, and 12 countries were lower than 50 in scores, while the scores of all the countries were normally distributed on the whole (Fig. 2B). South Africa performed best in the source of the infection indicator and ranked first in the height subindicators, along with scores above average in 22/28 subindicators. This suggests that OH initiatives for zoonoses, including capacity in surveillance and research activities, are being successfully implemented in this country [21, 35]. Mauritius ranked second behind South Africa for overall scores for zoonoses, with good performances in 3/5 indicators including the selected zoonotic case studies (rank 1), source of infection (rank 2), and targeted population (rank 3). Such performance aligns with the results following implementations of the strategic partnership for health security and emergence preparedness by the country in collaboration with the WHO and other international organizations [35]. Most had inconsistent performances in different indicators and subindicators, suggesting the implementation of some components of the OH approach to zoonoses. For example, Mali, Togo, and Sierra Leone ranked first, fifth, and twenty-sixth for overall scores, respectively. Remarkably, they received the highest score (first rank) for indicators of source of infection (Mali), route of transmission (Togo), and targeted population (Sierra Leone). These findings are consistent with (i) the OH project recently established in collaboration with the Swiss Tropical and Public Health Institute to tackle sources of zoonotic infections, e.g., as rabies, in Mali [20]; (ii) improvement in cross-border preparedness and response to zoonotic diseases in Togo [36]; and (iii) the establishment of national multisectoral coordination and collaboration mechanisms to prevent, detect, and respond to public health threats in Sierra Leone [37], especially after the bitter experience of the Ebola outbreak response, which served as an important catalyst for increased efforts to build the country’s capacity for health security and emergence preparedness. Furthermore, Benin ranked first in three subindicators and a number of height subindicators obtained above average scores, reflecting the country’s better performance in indicators of route of transmission and in zoonotic case studies than that of South Africa. This suggests that, despite its lowest overall score for zoonoses, Benin performed better for zoonotic disease control, especially in responding better to tuberculosis, COVID-19, echinococcosis, leishmaniasis, and rabies than South Africa did.

Results from this study provided OH performance for zoonoses and promoted awareness of OH by providing a reference in OH practice, research gaps and international assistance for sub-Sahara African countries. In addition, this study revealed an important finding where the governments of the 11 countries that were excluded are encouraged to direct more resources in the holistic application of the One Health approach for zoonoses so that their performance can be assessed in future. However, this work based on the OHIZ framework and official databases from authoritative organizations, which might be restrictive for the selection of the indicators within the scope of the framework.

In the last two decades, human beings have suffered from zoonoses. Zoonoses prevention and control had issues such as cross-border transmission and multidisciplinary integration. The OH approach provides an opportunity to overcome these challenges. In addition, the development of OH between countries needs to be synchronized. The OH concept has been raised at the beginning of the twenty-first century [38, 39] and has gained much more attention in recent years [40]. However, OH practices are still ignored at both the government and local levels, a cohesive network able to receive and act on early warnings at different levels is missing. The potential challenges that the OH approach will encounter when being implemented worldwide would be the establishment of efficient interdepartmental collaboration mechanisms and multidisciplinary platforms to support governance capacity in surveillance and research activities. Therefore, national and regional multisectoral coordination and collaboration mechanisms between medical doctors, veterinarians, public health experts, and food quality inspectors are needed to improve detection and responses to the public health matters holistically.

## Conclusions

Indicators to assess OH performance related to zoonoses are manifold, yet they are still not seemingly being embraced in developing countries, especially in sub-Saharan Africa, where zoonoses have the greatest impact. Findings from this study provide preliminary research information in advancing knowledge of the evidenced risks to strengthen OH strategies for effective control of zoonoses and to support prevention of a next zoonotic event.

## Supplementary Information

**Additional file 1. **Sources of data for the indicator sets used for zoonoses OHi.

## Data Availability

The full study protocol and the datasets, are available, following manuscript publication, upon request from the corresponding author Kokouvi Kassegne (kassegnek@sjtu.edu.cn).

## Abbreviations

AIDS: Acquired immune deficiency syndrome
BDI: Burundi
BEN: Benin
BFA: Burkina Faso
BWA: Botswana
CAF: Central African Republic
CCV: Cases of COVID-19
CIV: Cote d’Ivoire
CMR: Cameroon
COD: Democratic Republic of Congo
COVID-19: Novel coronavirus disease
CPV: Cabo Verde
DALYs: Disability-adjusted life years
DTT: Detection
ETH: Ethiopia
FAHP: Fuzzy analytical hierarchy process
FAO: United Nations’ Food and Agriculture Organization
GAB: Gabon
GBD: Global burden of disease
GHA: Ghana
GHDx: Global health data exchange
GHS: Global health security
GIN: Guinea
GOHI: Global One Health index
HDE: Human DALYs of echinococcosis
HDL: Human DALYs of leishmaniasis
HDR: Human DALYs of rabies
HDT: Human DALYs of tuberculosis
HPZ: Health promotion for zoonoses
IMS: Inhabitants below 5 m above sea level
KEN: Kenya
LBR: Liberia
LSO: Lesotho
MDG: Madagascar
MERS: Middle East respiratory syndrome
MLI: Mali
MOZ: Mozambique
MRT: Mauritania
MUS: Mauritius
MWI: Malawi
NAM: Namibia
NER: Niger
NGA: Nigeria
NPA: Natural protected areas
OH: One Health
OHHLEP: One Health high-level expert panel
OHIZ: One Health index on zoonoses
OIE: World Organization for Animal Health
PCI: Population coverage and cost of interventions
RWA: Rwanda
SARS: Severe acute respiratory syndrome
SDN: Sudan
SEN: Senegal
SLE: Sierra Leone
SNT: Sanitation
SVR: Surveillance and response
SYC: Seychelles
TCD: Chad
TGO: Togo
TZA: Tanzania
UGA: Uganda
UNEP: United Nations Environment Programme
VNR: Vaccination regulation
VRI: Vector and reservoir interventions
WB: World Bank
WHO: World Health Organization
ZAF: South Africa
ZAM: Zambia
ZWE: Zimbabwe
