# Supplementary material for: Assessment of performance for a key indicator of One Health: evidence based on One Health index for zoonoses in Sub-Saharan Africa
Source: Infect Dis Poverty. 2022 Oct 22;11:109. doi: 10.1186/s40249-022-01020-9 (PMC9588233; doi:10.1186/s40249-022-01020-9)
Supplement: Supplementary file 1 — Additional file 1. Sources of data for the indicator sets used for zoonoses OHi. [file 40249_2022_1020_MOESM1_ESM.docx]

| **Sources of data for the indicator sets used for zoonoses OHi** | | |
| --- | --- | --- |
| **Sub-indicator** | **Dataset** | **Source of data** |
| Strategy and Regulation  Surveillance and Response  Sanitation | National Guideline for Surveillance/Control  National Legislation on Animal Reservoirs  Zoonoses Capacity Score  General Surveillance  Vector Control  Wildlife Reservoirs Control  Basic Sanitation Services | Global Health Security Index  Global Health Security Index  World Health Organization  OIE-WHAIS  OIE-WHAIS  OIE-WHAIS  World Bank |
| Detection  Vector and Reservoir Interventions | Laboratory Testing for Zoonotic Reservoirs (Vectors and Animals)  Policy Adoption of Insecticide-Treated Mosquito Nets  Policy Adoption of Indoor Residual Spraying  Prevention Chemotherapy Coverage of Zoonoses | Global Health Security Index  World Health Organization  World Health Organization  World Health Organization |
| Vaccination Regulation  Population Coverage and Cost of Interventions  Inhabitants below 5 Meters above Sea Level | Vaccination Strategy and Regulation Vaccination  Proportion of Population Having Basic Drinking Water and Sanitation Facilities  Costs Directed to Chemotherapy/Vaccination of Humans  Number of Inhabitants below 5 Meters above Sea Level | Global Health Security Index  World Bank  World Health Organization  World Bank |
| Health Promotion for Zoonosis  Natural Protected Areas | Legislation of Zoonosis Educational Activities  Prevention and Control of Zoonoses  National Plan for Zoonoses Vaccine  Zoonotic Events and Human-Animal interface  Early Warning of Zoonoses  Emergency/Surveillance System  Proportion of Natural Protected Areas | World Health Organization  World Health Organization  World Health Organization  World Health Organization  World Health Organization  World Health Organization  World Bank |
| Cases of COVID-19  Human DALYs of Echinococcosis  Human DALYs of Leishmaniasis  Human DALYs of Rabies  Human DALYs of Tuberculosis | COVID-19 Infections Number  Vaccination Coverage for COVID-19  Echinococcosis DALYS  Leishmaniasis DALYS  Rabies DALYS  Tuberculosis DALYS | World Health Organization  World Health Organization  Global Health Data Exchange  Global Health Data Exchange  Global Health Data Exchange  Global Health Data Exchange |

OIE-WAHIS, OIE World Animal Health Information System

For each sub-indicator, the best/worst value was set, then following equation was used for normalization:

$$S_{ij}=\left\{ \frac{\begin{matrix} 0 \\ X_{ij}-X_{worst,j} \end{matrix}}{\begin{matrix} X_{best,j}-X_{worst,j} \\ 100 \end{matrix}}\times100 \right.$$

where $S_{\mathrm{ij}}$ denotes the normalized score for j-th sub-indicator of i-th country; $X_{\mathrm{ij}}$ denotes the original values for j-th sub-indicator of i-th country; $X_{best,j}$ denotes the original values of best performance for j-th sub-indicator; $X_{worst,j}$ denotes the original values of worst performance for j-th sub-indicator.

The weighted sum of the scores of the sub-indicators was derived from the following equation to obtain the scores of the weight indicators:

$$S_{ih}=\sum_{1_{h}}^{m_{h}} S_{\mathrm{ij}_{h}}\times W_{j_{h}} , \sum_{1_{h}}^{m_{h}} W_{j_{h}}=1$$

where $S_{ih}$ denotes the score of the h-th weight indicator of i-th country; $m$ denotes the total number of the sub-indicators under h-th weight indicator; $j_{h}$ denotes the j-th sub-indicator under h-th weight indicator; $S_{\mathrm{ij}_{h}}$ denotes the score of$j_{h}$-th sub-indicator of i-th country; $W_{j_{h}}$ denotes the weight of $j_{h}$-th sub-indicator.
